# The Expression Profile of Genes Related to Carotenoid Biosynthesis in Pepper Under Abiotic Stress Reveals a Positive Correlation with Plant Tolerance

**DOI:** 10.3390/life14121659

**Published:** 2024-12-13

**Authors:** Tingli Wang, Qiaoyun He, Chenyuan Wang, Zhimin Li, Shitao Sun, Xiai Yang, Xiushi Yang, Yanchun Deng, Chunsheng Hou

**Affiliations:** 1 Institute of Bast Fiber Crops, Chinese Academy of Agricultural Sciences, Changsha 410205, China; 82101222191@caas.cn (T.W.); 82101221142@caas.cn (Q.H.); 82101225154@caas.cn (C.W.); sunshitao@caas.cn (S.S.); yangxiai@caas.cn (X.Y.); yangxiushi@caas.cn (X.Y.); 2 Graduate School, Chinese Academy of Agricultural Sciences, Beijing 100081, China

**Keywords:** sweet pepper, carotenoid, abiotic stresses, VDE, LCYE

## Abstract

In light of the increasingly adverse environmental conditions and the concomitant challenges to the survival of important crops, there is a pressing need to enhance the resilience of pepper seedlings to extreme weather. Carotenoid plays an important role in plants’ resistance to abiotic stress. Nevertheless, the relationship between carotenoid biosynthesis and sweet pepper seedlings’ resistance to different abiotic stresses remains uncertain. In this study, the carotenoid content in abiotic-stressed sweet pepper seedling roots was determined, revealing that carotenoid content was extremely significantly elevated by more than 16-fold under salt stress, followed by drought stress (8-fold), and slightly elevated by only about 1-fold under waterlogging stress. After that, *serine/threonine-protein phosphatase 2A* (*PP2A*) was found to be the suitable reference gene (RG) in sweet pepper seedling roots under different abiotic stresses by using RT-qPCR and RefFinder analysis. Subsequently, using *PP2A* as the RG, RT-qPCR analysis showed that the expression level of most genes associated with carotenoid biosynthesis was extremely significantly up-regulated in sweet pepper seedlings under salt and drought stress. Specifically, *violoxanthin deepoxidase* (*VDE*) was significantly up-regulated by more than 481- and 36-fold under salt and drought stress, respectively; *lycopene epsilon cyclase* (*LCYE*) was significantly up-regulated by more than 840- and 23-fold under salt and drought stress, respectively. This study contributes to a more comprehensive understanding of the carotenoid biosynthesis pathway serving as a major source of retrograde signals in pepper subjected to different abiotic stresses.

## 1. Introduction

Carotenoids are natural pigments found in most fruits and vegetables, plants, algae, and photosynthetic bacteria [1]. Plant carotenoids, mainly a class of tetraterpenoid compounds, are plant pigments that serve multiple functions, including acting as antioxidants, hormone precursors, colorants, and essential components of the photosynthetic apparatus [2,3,4]. They primarily comprise α-carotene, β-carotene, lutein, zeaxanthin, lycopene, and cycloflavin [5]. These compounds accumulate in nearly all types of plastids, not solely within chloroplasts, thereby being present in most plant organs and tissues, such as roots, fruits, flowers, tubers, and seeds, albeit often at trace levels in certain areas [4]. Additionally, carotenoids, serving as important precursors of phytohormones, such as a vitamin A, abscisic acid (ABA), and strigolactones (SLs), function as regulators in plant development and play an important role in plant resistance to abiotic stress [5]. For instance, the accumulation of β-carotene in Arabidopsis and Salicornia europaea can enhance plant tolerance in high-salt environments [6,7]. Increased β-carotene content promoted waterlogging tolerance in tomato [8]. Despite the comprehensive study of carotenoids’ roles in responding to different stresses in plants, our understanding of the regulatory molecular mechanism remains limited.

**Figure 1:**
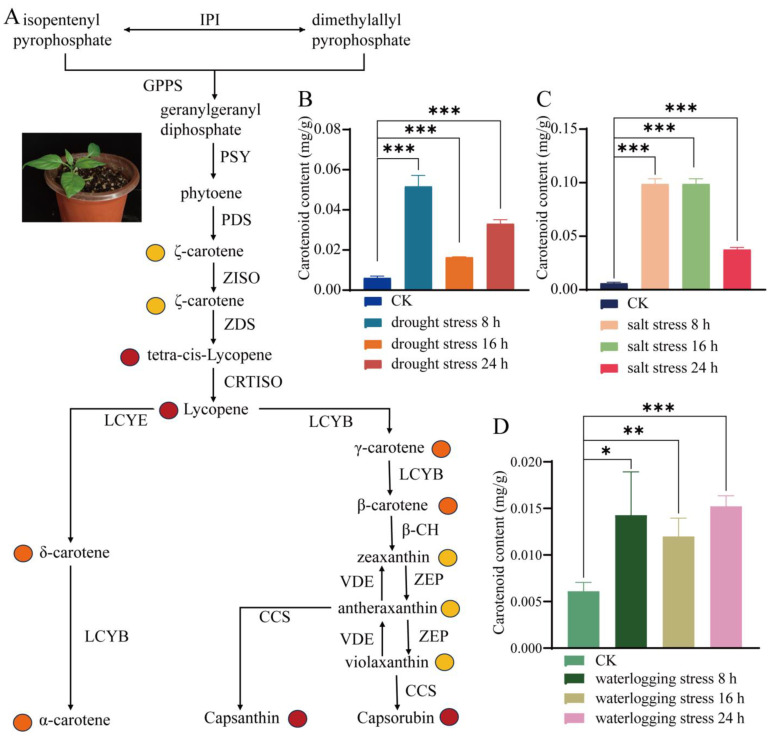
The different abiotic stresses that induce carotenoid biosynthesis in the root of pepper seedlings. (**A**) The carotenoid synthesis pathway in pepper. (**B**) The carotenoid content in the root of the pepper seedlings under drought stress. (**C**) The carotenoid content in the root of the pepper seedlings under salt stress. (**D**) The carotenoid content in the root of the pepper seedlings under waterlogging stress. The error bars: standard deviation (SD). The differences in carotenoid content between the control and stress-treated groups was calculated via a *t*-test using GraphPad Prism 8. *p* < 0.05 (*), *p* < 0.01 (**), and *p* < 0.001 (***) indicate significant, highly significant, and clearly highly significant differences at the 0.05, 0.01, and 0.001 levels. PSY: bifunctional 15-cis-phytoene synthase; PDS: 15-cis-phytoene desaturas; β-CH: beta-carotene hydroxylase; ZEP: zeaxanthin epoxidase; CCS: capsanthin/capsorubin synthase; ZISO: 15-cis-zeta-carotene isomerase; LCYE: lycopene epsilon cyclase; ZDS: zeta-carotene desaturase; CRTISO: carotenoid isomerase; GGPPS: geranylgeranyl pyrophosphate synthase; VDE: violaxanthin de-epoxidase; LCYB: lycopene beta cyclase.

As is well known, isopentenyl pyrophosphate (IPP) is synthesized by the mevalonic acid pathway (MVP) based on acetyl coenzyme A or the non-mevalonic acid pathway (MEP) based on phosphoenolpyruvate/phosphoglyceraldehyde. As shown in Figure 1A, IPP is involved in carotenoid biosynthesis as the active form of isopentadiene, which is converted to dimethylallyl pyrophosphate (DMAPP) by the action of isopentenyl pyrophosphate isomerase (IPI) [9]. The three IPP units are sequentially condensed onto DMAPP via geranylgeranyl diphosphate synthase (GGPPS) to produce the C20 precursor geranylgeranyl diphosphate (GGPP). The carotenoid biosynthesis pathway includes steps of condensation, desaturation/isomerization, hydroxylation, oxidation, and cyclization to produce various carotenoids and lutein [10,11]. Phytoene synthase (PSY) catalyzes the condensation of two GGPP molecules to produce the first carotenoid, 15-cis-octahydro lycopene [12,13]. Then, the 15-cis-octahydro lycopene is desaturated and isomerized by phytoene desaturase (PDS), ζ-carotene isomerase (ZISO), ζ-carotene desaturase (ZDS), and carotenoid isomerase (CRTISO), catalyzing the production of red all-trans lycopene [14]. Lycopene epsilon cyclase (LCYE) and lycopene beta cyclase (LCYB) subsequently cyclize all-trans lycopene to form symmetrical orange β-carotene and α-carotene in the β-β and ε-β branches, respectively [15,16,17]. β-Carotene is cyclized by β-carotene hydroxylase (β-CH), violoxanthin deepoxidase (VDE), the zeaxanthin epoxidase (ZEP), and capsanthin/capsorubin synthase (CCS) to produce the red colors of capsanthin and capsorubin [18,19]. Actually, numerous studies have demonstrated the pivotal role of genes related to carotenoid biosynthesis in mediating abiotic stress responses in plants [8,20,21]. The overexpression of the LCYB gene in sweet potato increases carotenoid content and enhances its tolerance to abiotic stress by affecting carotenoid and ABA biosynthesis [21]. TgLCYB1 regulated by TgWRKY22 improved the activities of antioxidant enzymes, thereby relieving waterlogging-induced oxidative damage [8]. Although there are existing comprehensive studies on the role of carotenoid production and the associated gene expression in the abiotic stress tolerance of species like sweet potatoes and tomatoes, little is known about peppers, a significant economic crop.

Currently, Capsicum spp. is the second most consumed vegetable worldwide, behind only tomato, and its market demand is increasing [22,23]. Drought stress, waterlogging stress, and salt stress are some of the main abiotic negative factors affecting pepper seedlings. In the present study, we initially investigated the changes in the carotenoid content of pepper under drought stress, waterlogging stress, and salt stress, and also quantitatively analyzed genes in the carotenoid biosynthesis pathway to gain a further understanding of the modulatory molecular mechanism underlying stress tolerance in pepper seedlings under different stresses.

## 2. Materials and Methods

### 2.1. Plant Materials and Growth Condition

Sweet pepper (*Capsicum annuum* L.) is one of the most important vegetable crops in the world due to its economic importance and the nutritional value of its fruits [24,25]. In this study, pepper (*C. annuum* Xiuli) seeds were germinated and sterilized in distilled water at 45 °C and sown in nutrient soil in the greenhouse of the National Bast Fiber Crops Germplasm Nursery at the Institute of Bast Fiber Crops, Chinese Academy of Agricultural Sciences (IBFC, CAAS, Changsha, China). Pepper seedlings were planted in a constant-temperature planting room under the following growing conditions: 24 ± l °C and 8 h/16 h (light/dark) alternating culture. Stress treatments were carried out using the pepper seedlings that had reached the four-leaf stage (Figure 1A). In consideration of prior experience, the maximum treatment time for seedlings was established as 24 h, with subsequent sampling intervals held to a consistent duration [26,27]. Salt stress: 12 pepper seedlings with uniform growth were selected and the roots were completely submerged at 2 cm in 200 mM sodium chloride (NaCl) for 8, 16, and 24 h. Drought stress: 12 pepper seedlings with uniform growth were selected and the roots were completely submerged at 2 cm in 20% PEG6000 for 8, 16, and 24 h. Waterlogging stress: 12 pepper seedlings with uniform growth were selected and the roots were completely submerged at 2 cm in sterile water for 8, 16, and 24 h. All samples were taken in triplicate, cooled rapidly in liquid nitrogen, and stored at −80 °C.

### 2.2. Quantitative Analysis of Carotenoid Content

Carotenoids have a special absorption peak at 440 ± 10 nm, and their content can be detected at this wavelength after solvent extraction of plant samples. In this study, carotenoids were extracted from pepper using a plant carotenoid content assay kit (BOXBIO, Beijing, China; No: AKPL004) and their contents were determined according to the instructions. Absorbance values (A440) at 440 nm were determined by using a spectrophotometer, and the carotenoid content in the root was calculated according to the following formula: carotenoid content $\left(\mathrm{mg}/g\right)=\frac{0.04\times A_{440}\times D}{W}$ (D: dilution ratio; W: weight of samples). All of the tests were repeated three times.

### 2.3. Total RNA Extraction and cDNA Synthesis

Liquid nitrogen rapid grinding was used to crush pepper tissue, the total RNA was extracted from the samples by using Trizol according to the instructions, and the quantity and quality of the RNA samples were measured by using a NanoDrop 2000 spectrophotometer (NanoDrop Technologies, Thermo Scientific, Boston, MA, USA). RNA samples with absorbance A260/280 between 1.9 and 2.1 and A260/230 above 2.0 were used for subsequent experiments. Meanwhile, the purity of the total RNA was detected by 1% (*w*/*v*) agarose gel electrophoresis with two clear 28S/18S ribosomal RNA bands. After that, the cDNA was synthesized using the HiFi-Script gDNA Removal cDNA Synthesis Kit (CWBIO, Jiangsu, China) according to the instructions and stored in the refrigerator at −20 °C for the real-time reverse transcriptase–polymerase chain reaction (RT-qPCR).

### 2.4. The Primer Design of RT-qPCR for the Genes Related to Carotenoid Biosynthesis and the Corresponding Reference Genes (RGs) for Different Abiotic Stresses

The 12 genes related to the carotenoid biosynthesis pathway in pepper mainly included *GGPPS*, *PSY*, *PDS*, *ZISO*, *ZDS*, *CRTISO*, *LCYB*, *LCYE*, *β-CH*, *ZEP*, *VDE*, and *CCS* (Figure 1A). However, the combination of the most stable RGs does vary amongst individual cultivars of peppers in different stress conditions. Moreover, none of the aforementioned studies analyzed the expression stability of RGs under different abiotic stresses. Based on the published literature [28,29], the common candidate RGs were selected and evaluated. Specific primers were designed according to the sequences of single genes using the online software (Primer 3 blast https://blast.ncbi.nlm.nih.gov/tools/primer-blas, accessed on 6 January 2021) on the NCBI website. The main parameters were as follows: primer length of 19–28 bp, melting temperature of 60–62 °C, GC content of 50–60%, and amplification product size of 185–315 bp (Table 1).

### 2.5. RT-qPCR Analysis

RT-qPCR was performed using the QuantiTect SYBR Green RT-PCR kit (Qiagen, Hilden, Germany). All reactions were performed using a 96-well optical plate in a CFX 96 real-time PCR system (Bio-Rad, Hercules, CA, USA). The total reaction system consisted of 20 μL:1 μL of the cDNA sample, 0.5 μL of the forward and reverse primers (10 μM), 10 μL of SYBR quantitative real-time PCR premix, and 8 μL of ddH_2_O. The amplification reaction conditions were as follows: pre-denaturation at 95 °C for 2 min, denaturation at 95 °C for 15 s, and annealing at 60 °C for 30 s, for a total of 40 cycles. The melting curve with a good single peak for each gene was auto-generated afterward in the detection system. As indicators of non-specific amplification and gDNA contamination, no template control (NTC) and no reverse transcriptase (NRT) were used. Three replicates of each sample were made.

### 2.6. Reference Gene Stability Analysis

RT-qPCR is an available and practical technique for assessing the transcript abundance of targeted genes in comparison with other quantitative methods, including Northern blotting, in situ hybridization, and RNA-seq technology [30,31]. Finding suitable reference genes (RGs) with consistently stable expression levels under a particular condition was crucial for RT-qPCR [32]. Based on the Ct (cycle threshold) values obtained from the RT-qPCR results, stability ranking of the candidate RGs was performed using geNorm [33], NormFinder [34], the Delta CT [35] method, and Bestkeeper [36]. The results of the four software analyses were re-scored and re-ranked using RefFinder [37] (https://blooge.cn/RefFinder/?type=reference, accessed on 2 October 2024) for a comprehensive analysis and evaluation of the stability of candidate RGs.

### 2.7. Statistical Analysis

Quantitative analysis of total carotenoid content was performed by using one-way analysis of variance (ANOVA), followed by Tukey’s multiple comparison test using GraphPad Prism 8 (GraphPad Software, San Diego, CA, USA). The expression level of the genes related to the carotenoid biosynthesis pathway was normalized by the qPCR data in Microsoft Excel using the 2^−ΔΔCT^ method, as done in previous studies [29], followed by a *t*-test using GraphPad Prism 8. *p* < 0.05 (*), *p* < 0.01 (**), and *p* < 0.001 (***) indicate significant, highly significant, and clearly highly significant differences at the 0.05, 0.01, and 0.001 levels.

## 3. Results

### 3.1. Carotenoid Accumulation Promoted Abiotic Stress Tolerance in the Sweet Pepper Seedlings

As is well known, roots are highly sensitive to changes in their surrounding environment, and root system responses to stresses such as salinity and drought can be very dynamic and complex in nature [38]. The carotenoid content of the roots of pepper seedlings subjected to different abiotic stresses (drought stress, waterlogging stress, and salt stress) was examined. Under the drought stress condition, compared to the CK group (0.2 mg/g) (0 h), the carotenoid content significantly increased more than 8 times, 2 times, and 5 times at 8 h, 16 h, and 24 h after 20% PEG6000 treatment, respectively (*p* < 0.001) (Figure 1B). Likewise, for the salt stress condition, the carotenoid content significantly increased more than 16 times, 15 times, and 6 times at 8 h, 16 h, and 24 h after 200 mM NaCl treatment, respectively (*p* < 0.001), in comparison with the CK group (Figure 1C). Meanwhile, the carotenoid content slightly increased more than 1.4 times, 1.0 times, and 1.5 times at 8 h, 16 h, and 24 h after sterile water treatment (waterlogging stress), respectively (*p* < 0.05) (Figure 1D). These results suggest that carotenoid played an important role in resisting abiotic stress in the sweet pepper seedlings.

### 3.2. Identification of RGs for Investigation of Transcriptomic Basis of Carotenoid Biosynthesis in Sweet Pepper Seedlings Under Different Stresses

RGs are required to normalize the expression level of target genes, and RT-qPCR is a readily available and useful technique for evaluating the transcript abundance of target genes [30,31,32]. In order to comprehend the critical role of genes related to carotenoid biosynthesis in mediating abiotic stress responses in pepper seedlings, finding suitable RGs is necessary for assessing the transcript abundance of target genes by RT-qPCR.

#### 3.2.1. Selection of Candidate RGs and Verification of Primers’ Specificity

**Figure 2:**
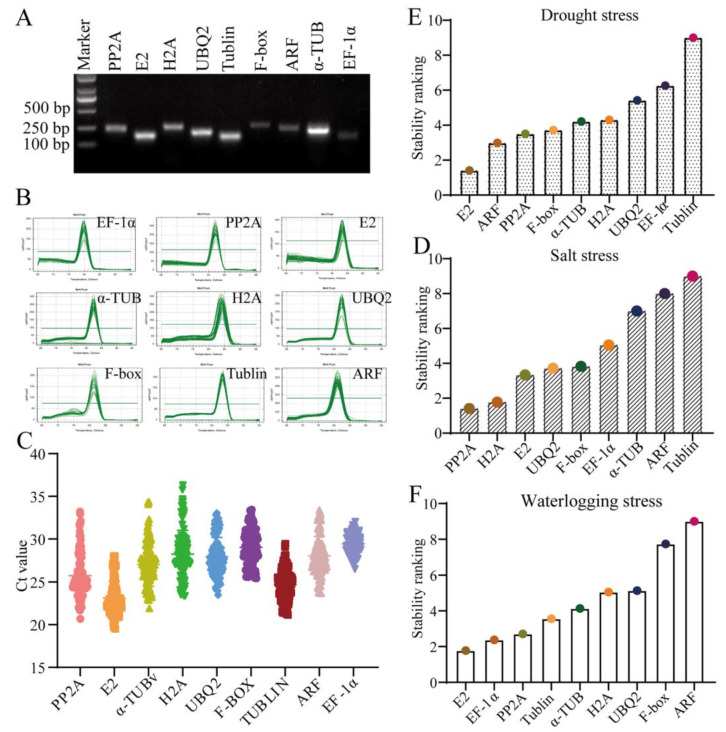
The identification of reference genes (RGs) for the investigation of the transcriptomic basis of carotenoid biosynthesis in pepper under different stresses. (**A**) Agarose gel electrophoresis of PCR products of the candidate RGs. (**B**) The RT-qPCR melting curve of the candidate RGs. (**C**) The distribution of Ct values of the candidate RGs. (**D**) RefFinder analysis of the stability of candidate RG expression in drought-stressed hot pepper seedlings. (**E**) RefFinder analysis of the stability of candidate RG expression in salt-stressed hot pepper seedlings. (**F**) RefFinder analysis of the stability of candidate RG expression in waterlogging-stressed hot pepper seedlings. *E2*: *ubiquitin-conjugating enzyme E2-17 kDa*; *α-TUB*: *tubulin alpha chain*; *H2A*: *histone H2A*; *UBQ2*: *ubiquitin carboxyl-terminal hydrolase 6*; *F-box*: *putative F-box protein At1g49610*; *Tublin*: *tubulin alpha-2 chain*; *ARF*: *ADP-ribosylation factor 1-like 2*; *EF-1α*: *elongation factor 1-alpha*.

Based on the common stable RGs used in the plants [30,32], nine candidate reference genes, *serine/threonine-protein phosphatase PP2A* (*PP2A*), *ubiquitin-conjugating enzyme E2* (*E2*), *tubulin alpha chain* (*α-Tub*), *histone H2A* (*H2A*), *ubiquitin carboxyl-terminal hydrolase 6* (*UBQ2*), *putative F-box protein At1g49610* (*F-box*), *tubulin alpha-2 chain* (*Tublin*), *ADP-ribosylation factor 1-like 2* (*ARF*), and *elongation factor 1-alpha* (*EF-1α*), were selected. The specificity of the primers was determined by agarose gel electrophoresis, which revealed that all of the RGs exhibited distinct and bright bands in the gel, with a size that corresponded to the predicted results (Figure 2A). Similarly, the specificity of the primers was also determined from the melting curves which exhibited a single peak (Figure 2B), indicating the absence of primer dimer formation and that the primers had strong specificity and could be employed for subsequent analyses.

#### 3.2.2. Analysis of the Expression Stability of RGs in Sweet Pepper Seedlings Under Different Stresses

The Ct (cycle threshold) values of RGs should range from 20 to 30 [39]. In the current study, the mean Ct values for the nine candidate RGs exhibited a range from 19.54 to 36.31. The Ct values of *E2,* which had the highest expression abundance, were 23.12 ± 1.98, while the Ct values of *H2A* and *EF-1α,* which had a relatively low expression abundance, were 28.23 ± 2.8 and 29.44 ± 1.2 (Figure 2C), suggesting that *H2A* and *EF-1α* may not be suitable RGs in the root of sweet pepper seedlings under different stresses. After that, RefFinder analysis, a comprehensive analysis ranking based on the stability analyses of geNorm, NormFinder, Bestkeeper, and delta CT, showed that *E2*, *ARF*, and *PP2A* demonstrated relatively stable expression in response to drought stress, while the expression of *tubulin* exhibited the lowest stability (Figure 2D). In the overall ranking of the expression stability of the RGs under salt stress, *PP2A* ranked first, while *tublin* ranked last (Figure 2E). In the root of flood-stressed pepper seedlings, *E2*, *EF-1α*, and *PP2A* were identified as stable genes, whereas *ARF* was determined to be the least effective RG (Figure 2F).

### 3.3. Expression Profile of Genes Related to Carotenoid Biosynthesis in Sweet Pepper Under Different Abiotic Stresses

Based on the above results, *PP2A* was found to be the most suitable RG in sweet pepper under drought stress, salt stress, and waterlogging stress in this study and was used to gain a further understanding of the expression profile of the genes related to carotenoid biosynthesis in sweet pepper under different abiotic stresses.

#### 3.3.1. The Changes in the Related Genes’ Expression Level Under Drought Stress

**Figure 3:**
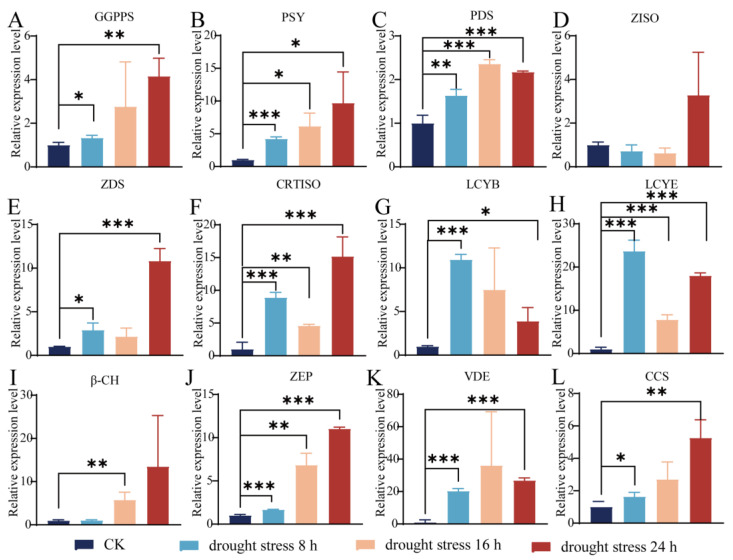
The expression profiles of the genes related to carotenoid biosynthesis in the roots of the pepper seedlings under drought stress. (**A**) *geranylgeranyl diphosphate synthase* (*GGPPS*). (**B**) *phytoene synthase* (*PSY*). (**C**) *phytoene desaturase* (*PDS*). (**D**) *ζ-carotene isomerase* (*ZISO*). (**E**) *ζ-carotene desaturase* (*ZDS*). (**F**) *carotenoid isomerase* (*CRTISO*). (**G**) *lycopene beta cyclase* (*LCYB*). (**H**) *lycopene epsilon cyclase* (*LCYE*). (**I**) *β-carotene hydroxylase* (*β-CH*). (**J**) *zeaxanthin epoxidase* (*ZEP*). (**K**) *violoxanthin deepoxidase* (*VDE*). (**L**) *capsanthin/capsorubin synthase* (*CCS*). The error bars: standard deviation (SD). The difference in the relative expression level of genes between the control and stress-treated groups was calculated via a *t*-test using GraphPad Prism 8. *p* < 0.05 (*), *p* < 0.01 (**), and *p* < 0.001 (***) indicate significant, highly significant, and clearly highly significant differences at the 0.05, 0.01, and 0.001 levels.

The 12 genes related to carotenoid biosynthesis in the roots of the sweet pepper seedlings subjected to drought stress (20% PEG6000 treatment) were examined by RT-qPCR (Figure 3). With the exception of the gene *ZISO*, there were notable alterations in the expression level of the remaining genes in the arid condition. Specifically, in comparison to the CK group (0 h), the expression level of *VDE* was significantly up-regulated more than 20 times, 36 times, and 26 times at 8 h, 16 h, and 24 h after 20% PEG6000 treatment, respectively (*p* < 0.001) (Figure 3K); the expression level of *LCYE* was significantly up-regulated more than 23 times, 7 times, and 18 times at 8 h, 16 h, and 24 h after 20% PEG6000 treatment, respectively (*p* < 0.001) (Figure 3H); the expression level of *CRTISO* was significantly up-regulated more than 8 times, 4 times, and 15 times at 8 h, 16 h, and 24 h after 20% PEG6000 treatment, respectively (*p* < 0.001) (Figure 3F).

#### 3.3.2. The Changes in the Related Genes’ Expression Level Under Salt Stress

**Figure 4:**
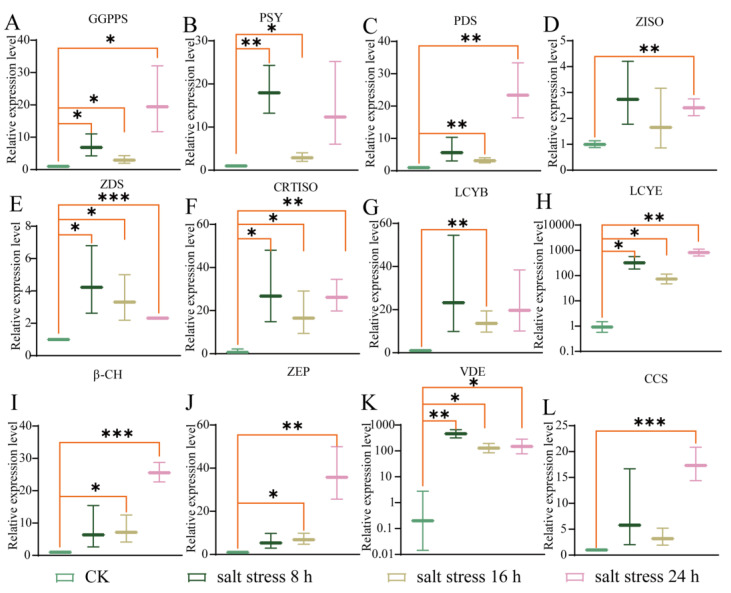
The expression profiles of the genes related to carotenoid biosynthesis in the roots of the pepper seedlings under salt stress. (**A**) *geranylgeranyl diphosphate synthase* (*GGPPS*). (**B**) *phytoene synthase* (*PSY*). (**C**) *phytoene desaturase* (*PDS*). (**D**) *ζ-carotene isomerase* (*ZISO*). (**E**) *ζ-carotene desaturase* (*ZDS*). (**F**) *carotenoid isomerase* (*CRTISO*). (**G**) *lycopene beta cyclase* (*LCYB*). (**H**) *lycopene epsilon cyclase* (*LCYE*). (**I**) *β-carotene hydroxylase* (*β-CH*). (**J**) *zeaxanthin epoxidase* (*ZEP*). (**K**) *violoxanthin deepoxidase* (*VDE*). (**L**) *capsanthin/capsorubin synthase* (*CCS*). The error bars: standard deviation (SD). The difference in the relative expression level of genes between the control and stress-treated groups was calculated via a *t*-test using GraphPad Prism 8. *p* < 0.05 (*), *p* < 0.01 (**), and *p* < 0.001 (***) indicate significant, highly significant, and clearly highly significant differences at the 0.05, 0.01, and 0.001 levels.

After that, the expression level of all genes associated with carotenoid biosynthesis in response to salt stress (200 mM NaCl treatment) was also examined and was found to be markedly elevated in comparison to the control group (Figure 4). In contrast to the drought environment, the alterations in the genes’ expression level in the salt environment were more obvious. For instance, compared to the CK group (0 h), the expression level of *VDE* was significantly up-regulated more than 481 times, 136 times, and 170 times at 8 h, 16 h, and 24 h after NaCl treatment, respectively (*p* < 0.001) (Figure 4K); the expression level of *LCYE* was significantly up-regulated more than 354 times, 78 times, and 840 times at 8 h, 16 h, and 24 h after NaCl treatment, respectively (*p* < 0.001) (Figure 4H). By comparison, the expression level of *ZDS* was significantly up-regulated only 4 times, 3 times, and 2 times at 8 h, 16 h, and 24 h after NaCl treatment, respectively (*p* < 0.05) (Figure 4E).

#### 3.3.3. The Changes in the Related Genes’ Expression Level Under Waterlogging Stress

**Figure 5:**
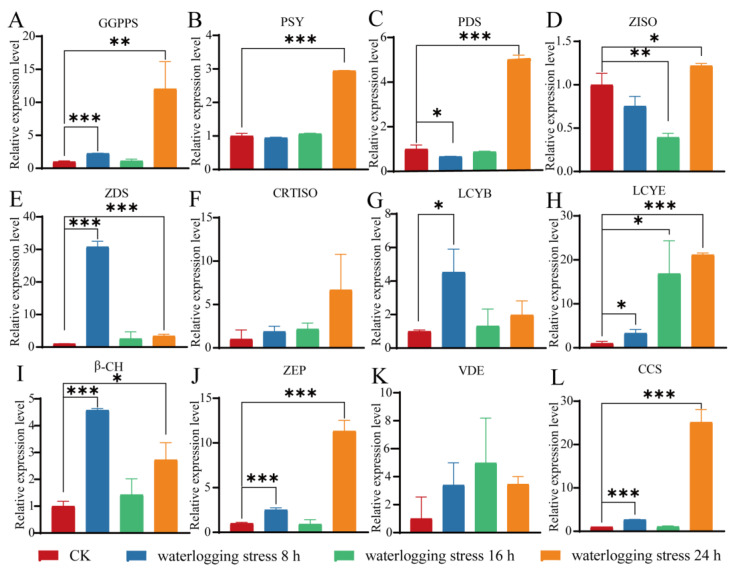
The expression profiles of the genes related to carotenoid biosynthesis in the roots of the pepper seedlings under waterlogging stress. (**A**) *geranylgeranyl diphosphate synthase* (*GGPPS*). (**B**) *phytoene synthase* (*PSY*). (**C**) *phytoene desaturase* (*PDS*). (**D**) *ζ-carotene isomerase* (*ZISO*). (**E**) *ζ-carotene desaturase* (*ZDS*). (**F**) *carotenoid isomerase* (*CRTISO*). (**G**) *lycopene beta cyclase* (*LCYB*). (**H**) *lycopene epsilon cyclase* (*LCYE*). (**I**) *β-carotene hydroxylase* (*β-CH*). (J) *zeaxanthin epoxidase* (*ZEP*). (**K**) *violoxanthin deepoxidase* (*VDE*). (**L**) *capsanthin/capsorubin synthase* (*CCS*). The error bars: standard deviation (SD). The difference in the relative expression level of genes between the control and stress-treated groups was calculated via a *t*-test using GraphPad Prism 8. *p* < 0.05 (*), *p* < 0.01 (**), and *p* < 0.001 (***) indicate significant, highly significant, and clearly highly significant differences at the 0.05, 0.01, and 0.001 levels.

Based on the slight change in carotenoid content after waterlogging stress, as expected, the impact of alterations in the expression levels of the related genes in pepper seedlings subjected to waterlogging stress exhibited a somewhat distinct pattern compared to the influence of drought and salt stress, and the expression level of the majority of genes exhibited faint discrepancies, except for in the 24-h treated pepper seedlings (Figure 5). Although the expression level of *LCYE* was still significantly up-regulated more than 3 times, 16 times, and 21 times at 8 h, 16 h, and 24 h after waterlogging treatment, respectively (*p* < 0.05) (Figure 5H), the expression level of *VDE* was slightly up-regulated (*p >* 0.05) (Figure 5K).

### 3.4. Expression Profile of Genes Related to Carotenoid Biosynthesis Under Abiotic Stresses Reveals Positive Correlation with Tolerance in Sweet Pepper

**Figure 6:**
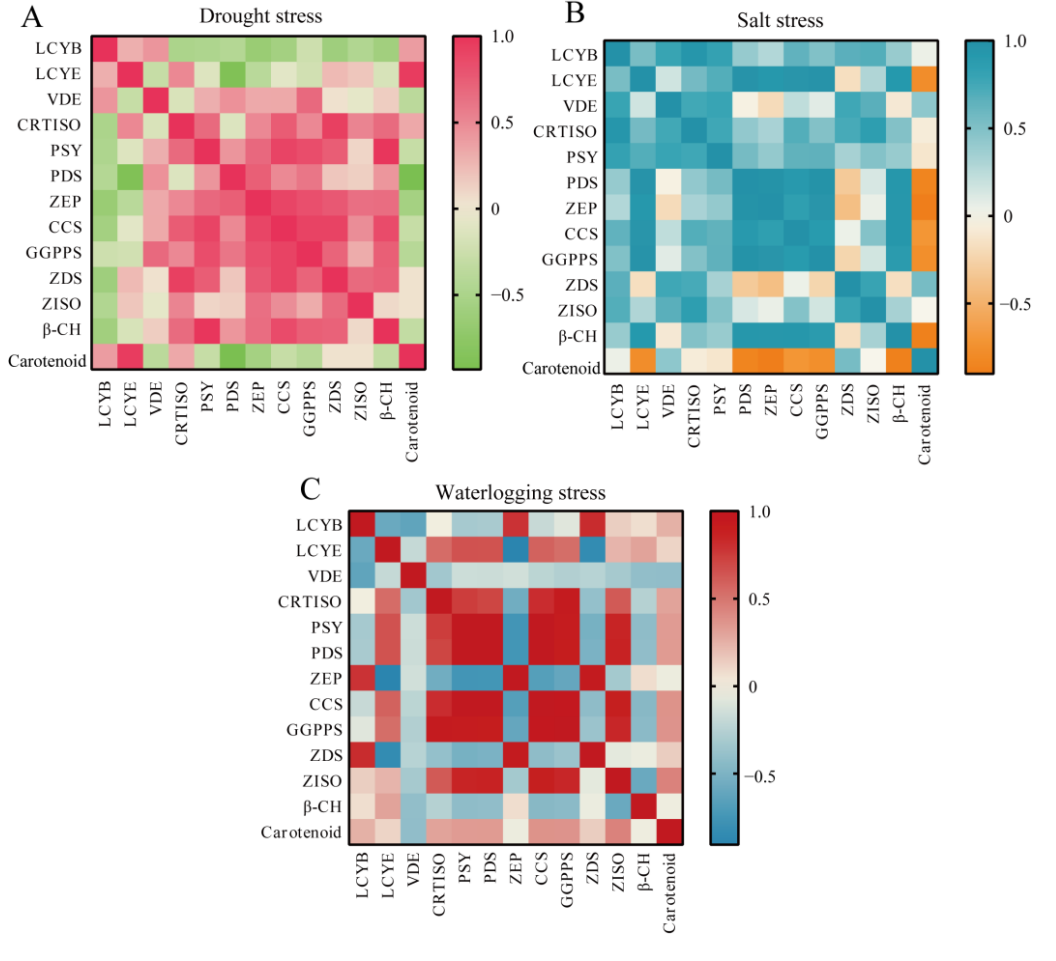
The correlation analysis of the expression level of genes related to carotenoid biosynthesis and carotenoid content in sweet pepper under different stresses. (**A**) The correlation analysis of the expression level of genes related to carotenoid biosynthesis and carotenoid content in pepper under drought stress. (**B**) The correlation analysis of the expression level of genes related to carotenoid biosynthesis and carotenoid content in pepper under salt stress. (**C**) The correlation analysis of the expression level of genes related to carotenoid biosynthesis and carotenoid content in pepper under waterlogging stress.

Based on the above results, the correlation analysis of the expression level of genes related to carotenoid biosynthesis and carotenoid content in sweet pepper under different stresses was performed (Figure 6). *LCYE* and *LCYB* were found to be positively correlated with drought tolerance in sweet pepper (*r* = 0.953, *p* < 0.05; *r* = 0.394) (Figure 6A); *ZDS* and *VDE* were found to be positively correlated with salt tolerance in sweet pepper (*r* = 0.542; *r* = 0.434) (Figure 6B); *CCS* and *GGPPS* were found to be positively correlated with waterlogging tolerance in sweet pepper (*r* = 0.373; *r* = 0.371) (Figure 6C).

## 4. Discussion

Red sweet peppers are renowned for their high antioxidant content and are widely consumed by people across the globe [40]; nevertheless, they are moderately salt-sensitive crops [41]. In addition, the effects of waterlogging stress on pepper plants include a reduction in growth and development, including photosynthesis and stomatal conductance [42]. The application of drought stress to peppers has also been found to result in a reduction in total chlorophyll content and a concomitant decrease in overall plant height [43]. Abiotic stresses on plants result in an increase in reactive oxygen species (ROS), which can be detrimental if not adequately eliminated; to this end, plants have evolved a range of antioxidant mechanisms to neutralize excess ROS by producing antioxidant active substances, such as carotenoids [44]. It has been demonstrated that carotenoids exhibit drought- resistance in both carrots and cotton [45,46], which is consistent with the present study in which the most significant difference in carotenoid content was observed after drought stress and salt stress, but not waterlogging stress, in the root of sweet pepper seedlings (Figure 1B,C). Also, carotenoid participates in salt stress tolerance in *Arabidopsis thaliana* and *Actinidia deliciosa* [47]. Upon waterlogging stress, the content of ABA (carotenoids serving as important precursors of ABA) was sharply decreased, alongside the decreased mRNA levels of the genes involved in the corresponding biosynthesis pathway, in the stem of *Myricaria laxiflora* and the root of *Prunus persica* [48,49], with this study suggesting that carotenoid biosynthesis is less involved in resistance to waterlogging stress.

Tolerance to different stresses in plants is achieved by not only pigment and hormone changes, but also alterations in gene expression, which can be monitored by quantifying the amounts of transcripts by RT-qPCR; the identification of optimal RGs for experiment normalization under different abiotic stresses was therefore necessary [50]. *Ubiquitin-conjugating protein* was found to be stably expressed in pepper under abiotic stress and hormonal treatments, and polyubiquitin-like protein was found to be stably expressed during pepper fruit development [28,29]. Similarly, in this study, the *ubiquitin-conjugating enzyme E2* and *PP2A* were identified as the most suitable RGs for pepper seedlings subjected to drought and waterlogging stress, and *PP2A* was also determined to be the most suitable reference gene for pepper seedlings treated with salt stress. Likewise, the *ubiquitin-conjugating enzyme E2* represents the optimal RG for *Platycladus orientalis* in response to abiotic stress [51]. *PP2A* was stably expressed in the roots and leaves of salt-stressed *Creeping bentgrass* [52,53]. *PP2A* is the most abundant protein phosphatase in eukaryotic cells and plays a vital role in plant oxidative stress signaling [54]. Previous work has indicated that the best ranked reference gene for salt stress was *PP2A* in Brassica napus [55]. Similarly, relatively stable expression was observed for *PP2A* in both roots and leaves subjected to salinity in bermudagrass [56], with the present study suggesting that *PP2A* is stably expressed in the roots of plants under different abiotic stresses.

The oxidation products of carotenoids can act as signals relaying that plants are subjected to stress, inducing changes in gene expression that enable them to adapt to extreme environments [57,58]. It has been reported that, associated with carotenoid biosynthesis genes, *DXR* plays a significant role in the regulation of salt stress and participates in multiple physiological responses in kiwifruit [59]; *PSY* plays an important role in response to salt stress conditions in *Daucus carota* [60], which is consistent with this study in which the expression level of all genes associated with carotenoid biosynthesis in response to salt stress was markedly elevated (Figure 4). Specifically, the expression level of *VDE* and *LCYE* was extremely significantly up-regulated in the root of sweet pepper seedlings under salt stress and drought stress (Figure 3 and Figure 4). It has been reported that overexpression of *VDE* improves drought-induced photo-damage in *Arabidopsis* [61], *LCYE* is a limiting enzyme for carotenoid accumulation [62], and carotenoid biosynthesis exhibits drought and salt stress tolerance in various plants [45,46,47]. Interestingly, *LCYE* was found to be significantly positively correlated with drought tolerance and *VDE* was found to be positively correlated with salt tolerance in sweet pepper (Figure 6). In addition, the expression level of *VDE* was found to be slightly up-regulated in seedling roots under waterlogging stress, which was consistent with the result of carotenoid content (Figure 1 and Figure 5). Meanwhile, *ZDS* was significantly up-regulated in the root of sweet pepper seedlings under waterlogging stress (Figure 5). As reported, *ZDS* plays an important role in plant resistance to saline–alkali stress and provides excellent resistance genes for the regulatory network of salinity stress response in apples [63]. These results suggest that the central role of the carotenoid biosynthesis pathway serves as a major source of retrograde signals in pepper subjected to different abiotic stresses, although further research is needed to elucidate the underlying mechanisms.

## 5. Conclusions

Overall, this study highlights the role of the carotenoid biosynthesis pathway in exhibiting drought resistance, salt resistance, and waterlogging resistance in the roots of sweet pepper. According to our findings, *PP2A* was found to be the most suitable RG in sweet pepper seedlings under various abiotic stresses. The genes associated with carotenoid biosynthesis, such as *VDE* and *LCYE*, are crucial for pepper seedling roots’ ability to respond to salt and drought stress. Although carotenoids mostly include α-carotene and β-carotene, it is still unknown which carotenoid in pepper is specifically responsible for abiotic stress resistance, and the role and molecular regulatory mechanism of carotenoid biosynthesis remain unexplored.

## Figures and Tables

**Table 1:** Primer sequences of candidate reference genes.

Genes	ID	Full Name	Primer Sequences (5′-3′)	Size (bp)
*PP2A*	LOC107843392	serine/threonine-protein phosphatase PP2A-5 catalytic subunit	F: CCGTGGTGCTGGATACACTT R: GGTTCTCCCCTTCTTGGAGC	255
*E2*	LOC107873556	ubiquitin-conjugating enzyme E2-17 kDa	F: TAAACTTTCAGGGTTTGGAGTTG R: TACACCTCCAGCATAAGGGC	189
*α-TUB*	LOC107867313	tubulin alpha chain	F: CAGCTAACAACTTTGCCCGT R: GTAAGGTTCATCCACAGAGGT	260
*H2A*	LOC107848286	histone H2A	F: AGCCTGTTTCCCGTTCTGTC R: TGTTTGGAAGAACACCGCCAT	294
*UBQ2*	LOC107850577	ubiquitin carboxyl-terminal hydrolase 6	F: AGAACCAGAAACCTCTGCCG R: ACCAATCAGCGTCGTCCTTT	228
*F-box*	LOC107880054	putative F-box protein At1g49610	F: GGACTTTGTGAACCGGACCT R: TGTCATCCCACAACACCGAG	315
*Tublin*	LOC107848102	tubulin alpha-2 chain	F: CCAGTGTTGCTGAGGTCTTCT R: TCGCCGTCGTCCAATTCA	185
*ARF*	LOC107875797	ADP-ribosylation factor 1-like 2	F: CGGAAAGGCAAAGCAAGAGT R: CTGAAGTGCCCACGGAAGAG	275
*EF-1α*	LOC107862188	elongation factor 1-alpha	F: AGAAAATGCAGTACCTTTCAAFT R: AAGAGCTTGGATGCCCTTCA	191
*PSY*	LOC107868281	bifunctional 15-cis-phytoene synthase	F: ATGTCTGTTGCCTTGTTATGG R: CCTGATTTCATGTTCTTGTAGAAGG	267
*PDS*	LOC107861625	15-cis-phytoene desaturase	F: AGCCAGGAGAATTCAGCCGC R: CGTCACCCTATCCGGCACAC	226
*β-CH*	LOC107863219	beta-carotene hydroxylase	F: GCACGAGTCACACCATAGACCAAG R: CGTGAACGAACATGTAGGCCATCC	188
*ZEP*	LOC107860302	zeaxanthin epoxidase	F: TGCACTTCATCCAATGACACCT R: GCCTCTGAAATGCACCTTGC	90
*CCS*	LOC107875664	capsanthin/capsorubin synthase	F: AGCACCCACATCAAAGCCAG R: GTGGTGAAGGGTCAACGCAA	260
*ZISO*	LOC107850257	15-cis-zeta-carotene isomerase	F: GGTGGGATTCTTGGTGTTGT R:GGTAAGGGAAAGCATTACAATCTC	135
*LCYE*	LOC107840923	lycopene epsilon cyclase	F: AACTTCCCTCCTCCCTATCCA R: GTTTGGTCAATTGGGTGTTTGA	135
*ZDS*	LOC107839468	zeta-carotene desaturase	F: TCGGATGAGTTGAGTCTTGTC R: ACTGAAGCGTGGCAGAATAGA	198
*CRTISO*	LOC107854534	carotenoid isomerase	F: TCCAACTTGGTTGGGTCTCT R: GGTCTCATATGATTCTTGCCCT	188
*GGPPS*	LOC107867046	geranylgeranyl pyrophosphate synthase	F: CGTCAGAGGAGCTCGGAAAA R: TTGCGCGAATCAAACCCTTC	151
*VDE*	LOC107850430	violaxanthin de-epoxidase	F: CGGTTTGCAGGGAAACAAGAAA R: AAGGAGTTTTGTGGGGTTTGT	159
*LCYB*	LOC107869983	lycopene beta cyclase	F:ATGGGTGGTCCTCTTCCAGT R: ATCGGCCACAAATCCTTCCA	209

## Data Availability

The data that support the findings of this study are available in this article.
